# Ciprofloxacin and levofloxacin attenuate microglia inflammatory response via TLR4/NF-kB pathway

**DOI:** 10.1186/s12974-019-1538-9

**Published:** 2019-07-18

**Authors:** Morena Zusso, Valentina Lunardi, Davide Franceschini, Andrea Pagetta, Rita Lo, Stefano Stifani, Anna Chiara Frigo, Pietro Giusti, Stefano Moro

**Affiliations:** 1 Department of Pharmaceutical and Pharmacological Sciences, University of Padua, Largo E. Meneghetti 2, 35131 Padua, Italy; 2 Present address: Selvita S.A., Park Life Science ul, Bobrzyńskiego 14, 30-348 Kraków, Poland; 3 Department of Neurology and Neurosurgery, Montreal Neurological Institute, McGill University, Montreal, QC H3A 2B4 Canada; 4 Unit of Biostatistics, Epidemiology and Public Health, Department of Cardiac, Thoracic and Vascular Sciences, University of Padua, Padua, Italy

**Keywords:** Neuroinflammation, Microglia, Fluoroquinolones, TLR4–MD-2 complex, Nuclear factor-κB, Pro-inflammatory cytokines

## Abstract

**Background:**

Neuroinflammation is the response of the central nervous system to events that interfere with tissue homeostasis and represents a common denominator in virtually all neurological diseases. Activation of microglia, the principal immune effector cells of the brain, contributes to neuronal injury by release of neurotoxic products. Toll-like receptor 4 (TLR4), expressed on the surface of microglia, plays an important role in mediating lipopolysaccharide (LPS)-induced microglia activation and inflammatory responses. We have previously shown that curcumin and some of its analogues harboring an α,β-unsaturated 1,3-diketone moiety, able to coordinate the magnesium ion, can interfere with LPS-mediated TLR4–myeloid differentiation protein-2 (MD-2) signaling. Fluoroquinolone (FQ) antibiotics are compounds that contain a keto-carbonyl group that binds divalent ions, including magnesium. In addition to their antimicrobial activity, FQs are endowed with immunomodulatory properties, but the mechanism underlying their anti-inflammatory activity remains to be defined. The aim of the current study was to elucidate the molecular mechanism of these compounds in the TLR4/NF-κB inflammatory signaling pathway.

**Methods:**

The putative binding mode of five FQs [ciprofloxacin (CPFX), levofloxacin (LVFX), moxifloxacin, ofloxacin, and delafloxacin] to TLR4–MD-2 was determined using molecular docking simulations. The effect of CPFX and LVFX on LPS-induced release of IL-1β and TNF-α and NF-κB activation was investigated in primary microglia by ELISA and fluorescence staining. The interaction of CPFX and LVFX with TLR4–MD-2 complex was assessed by immunoprecipitation followed by Western blotting using Ba/F3 cells.

**Results:**

CPFX and LVFX bound to the hydrophobic region of the MD-2 pocket and inhibited LPS-induced secretion of pro-inflammatory cytokines and activation of NF-κB in primary microglia. Furthermore, these FQs diminished the binding of LPS to TLR4–MD-2 complex and decreased the resulting TLR4–MD-2 dimerization in Ba/F3 cells.

**Conclusions:**

These results provide new insight into the mechanism of the anti-inflammatory activity of CPFX and LVFX, which involves, at least in part, the activation of TLR4/NF-κB signaling pathway. Our findings might facilitate the development of new molecules directed at the TLR4–MD-2 complex, a potential key target for controlling neuroinflammation.

**Electronic supplementary material:**

The online version of this article (10.1186/s12974-019-1538-9) contains supplementary material, which is available to authorized users.

## Background

Neuroinflammation is the complex immune response of the central nervous system (CNS). When sustained, neuroinflammation is a common denominator in the etiology and course of neurodevelopmental, neurodegenerative, and psychiatric disorders (e.g., Alzheimer disease, Parkinson disease, multiple sclerosis, motor neuron disease, autism spectrum disorder, schizophrenia, and depression) (for a review, see [1]). Cellular (e.g., microglia and astrocytes) and molecular (e.g., cytokines, complement, and pattern-recognition receptors) immune components act as key regulators of neuroinflammation. Their dysregulated activity results in an inappropriate immune response that can lead to tissue damage and affect CNS functions [2]. Aberrantly activated microglia, the resident macrophages of the CNS, are the predominant source of a plethora of inflammatory and cytotoxic mediators, which have been implicated in neuronal dysfunctions and brain damage [3, 4].

Microglia express a wide range of receptors, including toll-like receptors (TLRs), a subfamily of pattern-recognition receptors that recognize invading pathogens and endogenous harmful stimuli to induce innate and adaptive immune responses [5]. Among TLRs, TLR4 is the major lipopolysaccharide (LPS) receptor [6, 7]. When expressed on the cellular membrane, TLR4 exists as a complex with the co-receptor myeloid differentiation protein-2 (MD-2), which is essential for LPS recognition by TLR4–MD-2 complex [8, 9]. Binding of LPS causes the TLR4–MD-2 complex dimerization [10], which results in the activation of downstream mediators, including the transcription factor nuclear factor (NF)-κB, which increases the production of pro-inflammatory molecules, such as cytokines (e.g., tumor necrosis factor (TNF)-α, interleukin (IL)-1β, and IL-6), chemokines, enzymes, and reactive oxygen and nitrogen species [11]. Thus, targeting microglia and TLR4–MD-2 complex activation is gaining increasing interest as a potential therapeutic or preventive strategy for the treatment of CNS disorders.

Curcumin, the major bioactive component extracted from the rhizome of *Curcuma longa*, has been extensively studied for its wide range of biological activities, including anti-inflammatory properties. Considering that curcumin crosses the blood-brain barrier maintaining its biological activity [12, 13], it has been proposed for the treatment of various neuroinflammatory and neurodegenerative conditions. Curcumin is a highly pleiotropic molecule that interacts with numerous molecular targets [14, 15]. Several studies have demonstrated that curcumin attenuates inflammatory response via TLR4 pathway. A recent study has shown that curcumin administration may improve neuroinflammatory outcomes by reducing microglia/macrophage activation and neuronal apoptosis through a mechanism involving the TLR4/MyD88/NF-κB signaling pathway in microglia/macrophages [16]. Furthermore, in vitro, curcumin inhibits the homodimerization of TLR4, which is required for the activation of downstream signaling pathways of this receptor [17, 18]. In addition, recently, we have shown that the anti-inflammatory activity of curcumin and some of its derivatives having an α,β-unsaturated 1,3-diketone moiety is associated with the ability to coordinate Mg^2+^, affecting the proper assembly of the TLR4–MD-2–LPS ternary complex [18].

In recent years, many antibacterial agents, including fluoroquinolones (i.e., 7-fluoro-4-oxo-1,4-dihydroquinoline-3-carboxylic acid) (FQs), one of the most important and commonly prescribed classes of synthetic antibiotics [19], have been shown to exert immunomodulatory activities by decreasing the production and release of inflammatory-associated cytokines, both in vitro and in vivo, in addition to their classical antimicrobial activity [20–25]. Several underlying mechanisms of the immunomodulatory activity of FQs have been proposed, including the inhibition of phosphodiesterase and transcription factors, such as activator protein-1, nuclear factor of activated T cells, nuclear factor-IL-6, and NF-κB [22]. However, to date, the precise cascade of events and, most important, the primary target for the anti-inflammatory properties of FQs have not been defined. Although the interference of FQs with cellular receptors, such as TLRs, has been hypothesized [21, 26], there is still no evidence showing any interactions of these drugs with TLRs or other cell membrane receptors associated with inflammatory signaling.

The aim of the present study was to investigate the role of TLR4 signaling in the anti-inflammatory activity of FQs. By molecular modeling simulations, we first characterized the putative binding mode of five FQs [ciprofloxacin (CPFX), levofloxacin (LVFX), moxifloxacin (MXFX), ofloxacin (OFX), and delafloxacin (DLFX)] to the TLR4–MD-2 complex. Next, we examined the effect of CPFX and LVFX, two of the most largely prescribed antibiotics, on LPS-induced microglia activation and sought to identify their molecular targets in the TLR4 signaling pathway (i.e., TLR4–MD-2 complex and NF-κB activation), using primary microglial cells and Ba/F3 cells, a murine interleukin-3 dependent pro-B cell line [27]. We found that CPFX and LVFX reduced the release of pro-inflammatory cytokines by LPS-activated microglia. They also inhibited LPS-induced activation of NF-κB, one of the major transcription factors implicated in TLR4 signaling. Finally, we showed that the two FQs prevented the engagement of LPS to TLR4–MD-2 complex and its dimerization, indicating that the binding between LPS and the receptor complex is the target for the anti-inflammatory properties of CPFX and LVFX.

## Methods

### Materials

Unless otherwise specified, all reagents were from Sigma-Aldrich (Milan, Italy). Tissue culture media, antibiotics, and fetal calf serum (FCS) were obtained from Life Technologies (San Giuliano Milanese, Italy). LPS (Ultra-Pure LPS-EB from *Escherichia coli*, 0111:B4 strain) and biotinylated LPS (LPS-EB Biotin from *Escherichia coli*, 0111:B4) were purchased from InvivoGen (InvivoGen Europe, Toulouse, France); these ultrapure preparations only activate the TLR4 pathway. Primary antibodies included mouse anti-p65 (NF-κB p65, Santa Cruz Biotechnology, Santa Cruz, CA, USA), mouse and rabbit anti-GFP (#NB600-597 and #NB600-308; Novus, Littleton, CO, USA), and mouse anti-Flag (#F3165; Sigma-Aldrich). AlexaFluor 555 and horseradish peroxidase (HRP)-conjugated secondary antibodies were from Molecular Probes (Rockville, MD, USA) and Bio-Rad Laboratories (Hercules, CA, USA), respectively. Falcon tissue culture plastic-ware was purchased from BD Biosciences (SACCO srl, Cadorago (CO), Italy).

### Target structures

The crystallographic structure of the human TLR4-human MD-2-*E*. *coli* LPS ternary complex was retrieved from the Protein Data Bank (PDB code: 3FXI) [10]. Assessment of crystallographic structure quality was performed with the Structure Preparation tool of the Molecular Operation Environment program (MOE, version 2014.09; Chemical Computing Group Inc., 1010 Sherbooke St. West, Suite #910, Montreal, QC, Canada, H3A 2R7, 2015). Critical structural issues (such as missing or poorly resolved atomic data, anomalous topological properties present in amino acid units, as well as anomalous bonding patterns of non-amino acid units) were fixed when necessary. Hydrogen atoms were added and their appropriate protonation state fixed using the Protonate3D tool as implemented in the MOE program. To minimize contacts between hydrogen atoms, the structures were subjected to Amber99 force field [28] minimization until the root-mean-square (rms) of the conjugate gradient was < 0.1 kcal mol^−1^ Å^−1^ keeping the heavy atoms fixed at their crystallographic positions.

### Molecular docking protocol

CPFX, LVFX, MXFX, OFX, and DLFX structures were built using the “Builder” module of MOE, and each compound was docked into the presumptive binding sites (LPS binding site) using flexible MOE-Dock methodology. The purpose of MOE-Dock is to search for favorable binding configurations between a small, flexible ligand and a rigid macromolecular target. Searching is conducted within a user-specified 3D docking box, using the “tabù search” [29] protocol and the MMFF94 force field [30]. Charges for ligands were imported from the MOPAC program [31] output files. MOE-Dock performs a user-specified number of independent docking runs (50 in the present case) and writes the resulting conformations and their energies to a molecular database file. The resulting ligand/protein complexes were subjected to MMFF94 all-atom energy minimization until the rms of conjugate gradient was < 0.1 kcal mol^−1^ Å^−1^. GB/SA approximation [32] has been used to model the electrostatic contribution to the free energy of solvation in a continuum solvent model. The interaction energy values were calculated as the energy of the complex minus the energy of the ligand, minus the energy of the protein: Δ*E*_inter_ = *E*_(complex)_ − (*E*_(L)_ + *E*_(Protein)_).

### Cell cultures

Sprague-Dawley rats (CD strain) were maintained under controlled temperature and humidity, with free access to water and food on a 12-h light/dark cycle (lights on at 7:00 am). Animal-related procedures were performed in accordance with EU guidelines for the care and use of laboratory animals and those of the Italian Ministry of Health (D.L. 26/2014), and were approved by the Institutional Review Board for Animal Research (Organismo Preposto al Benessere Animale, OPBA) of the University of Padua and by the Italian Ministry of Health (protocol number 958/2016-PR). Pregnant females were monitored for the parturition day, which was counted as postnatal day 0 (PN0). Primary microglial cells were isolated from mixed glial cell cultures prepared from cerebral cortex of PN1 rat pups, as previously described [33], with slight modifications. Briefly, when mixed glial cultures reached confluence (typically 7–10 days after isolation), microglia were separated from the astroglial monolayer by shaking (200 rpm for 1 h at 37 °C), re-suspended in high-glucose Dulbecco’s modified eagle medium (DMEM) supplemented with 2 mM l-glutamine, 10% heat-inactivated FCS, 100 units/ml penicillin, 100 μg/ml streptomycin, and 50 μg/ml gentamicin (growth medium), and plated on poly-l-lysine-coated (10 μg/ml) plastic wells at a density of 1.50 × 10^5^ cells/cm^2^. Cells were allowed to adhere for 45 min and then washed to remove non-adhering cells. Cultures obtained using the shaking procedure generated 97% microglia immunopositive to a primary polyclonal antibody against ionized calcium binding adaptor molecule 1 (1:800, Wako Pure Chemical Industries, Ltd., Osaka Japan), a marker for microglia cell types [34, 35].

Murine Ba/F3 cells, an IL-3 dependent pro-B cell line, stably expressing human TLR4-GFP, human TLR4-Flag, human MD-2-Flag, and human CD14 were kindly provided by Dr. Kensuke Miyake (University of Tokyo, Japan). Cells were cultured in RPMI1640 medium, supplemented with 10% FCS, 100 μM 2-mercaptoethanol, and recombinant murine IL-3 (~ 70 U/ml) [27]. Cells were maintained at 37 °C in a humidified atmosphere containing 5% CO2/95% air.

### Cell viability assays

Microglial cell viability was evaluated by three colorimetric methods utilizing the metabolic dye 3-(4,5-dimethylthiazol-2-yl)-2,5-diphenyltetrazolium bromide (MTT) [36], the protein-binding dye sulforhodamine B (SRB) [37, 38], and the Trypan blue assay [39]. Cells were seeded in poly-l-lysine coated 96-well plates (50,000 cells/well) in growth medium and allowed to adhere overnight. Growth medium was replaced with serum-free medium 2 h before pretreatment for 1 h with increasing concentrations of CPFX and LVFX (50–200 μg/ml) followed by stimulation with 100 ng/ml Ultra-Pure LPS-EB for an additional 6 h. After the incubation, the medium was removed, and the cells incubated with MTT (0.18 mg/ml) for 4 h at 37 °C. The formazan crystals were solubilized with DMSO. The plates were then analyzed on a microplate reader (Victor2 Multilabel Counter, Wallac, Cambridge, MA, USA) using a test wavelength of 570 nm and a reference wavelength of 630 nm. For the SRB assay, after 6 h incubation with the tested compounds, the medium was replaced with cold 10% trichloroacetic acid, and plates were incubated for 1 h at 4 °C. Following this fixation step, cells were stained with 0.4% SRB and left at room temperature for 30 min. The bound protein stain was solubilized with 10 mM Tris base. The optical density was then measured at 570 nm with a microplate reader. After treatment, cells were washed, detached with 0.25% trypsin-0.2% EDTA, and resuspended in 0.4% Trypan blue at 1:1 ratio in serum-free complete medium. Cells were counted with a Burker chamber hemocytometer. Results are expressed as percentage viability relative to untreated cultures.

### Cytokine determination

Primary microglia were pretreated for 1 h with increasing concentrations of CPFX and LVFX and then stimulated with 100 ng/ml Ultra-Pure LPS-EB for an additional 6 h. At the end of incubation, culture medium was collected and IL-1β and TNF-α assayed using commercially available ELISA kits (Antigenix America, Huntington Station, NY, USA), according to the manufacturer’s instructions. Cytokine concentrations (pg/ml) in the medium were determined by reference to standard curves obtained with known amounts of IL-1β or TNF-α, and the results were expressed as percentage relative to LPS-stimulated cultures.

### Immunofluorescence and image analysis

Microglia were grown on coverslips in 12-well plates and pretreated for 1 h with CPFX and LVFX and then stimulated with 100 ng/ml Ultra-Pure LPS-EB for an additional 90 min. Cells were fixed with 4% paraformaldehyde (pH 7.4, for 15 min at room temperature) and subsequently non-specific staining was blocked by incubating with 5% normal goat serum/0.1% Triton X-100 in PBS for 1 h at room temperature. Cells were then incubated sequentially with a mouse anti-p65 primary antibody (NF-κB p65, 1:500) for 2 h, followed by Alexa Fluor 555-conjugated secondary antibody (1:1000) for 1 h in the above blocking solution. Cells were thoroughly washed between steps with PBS [13]. Immunostaining control included omission of the primary antibody. Nuclei were stained with 4,6-diamidino-2-phenylindole (DAPI; 0.1 μg/ml) and coverslips were mounted on microscope slides with Fluoromount-G mounting medium (Fisher Scientific, Milan, Italy). Fluorescent images were captured with a confocal laser-scanning microscope (Zeiss LSM 800; Carl Zeiss AG, Germany) and microscope settings were kept constant for all images. For each image, three z-stacks (50 μm optical section, 1.5 μm total Z-span) were acquired with a × 63, NA 1.4, oil-immersion objective. All images were taken from the same plane, considering the center of nuclei as the central plane for z-stack. ImageJ software (National Institutes of Health, Bethesda, MD, USA) was used to flatten each z-stack image into a single image, representing the sum of the contributes from each focal plane. Fluorescence emission intensity of single cells was profiled using ImageJ software [18]. To quantitatively evaluate subcellular distribution of p65 subunit, the relative staining intensities in the nucleus and cytoplasm were monitored from five random fields for each condition from three independent experiments. Cytoplasmic and nuclear fluorescence intensities were calculated using ImageJ software and are expressed as a percentage of nuclear and cytoplasmic NF-κB p65 subunit.

### Immunoprecipitation and Western blotting

Immunoprecipitation experiments were performed using Ba/F3 cells stably expressing human TLR4-GFP, human TLR4-Flag, human MD-2-Flag, and human CD14. Ba/F3 cells (80 × 10^6^ cells/condition) were pretreated for 1 h with CPFX or LVFX (500 μg/ml) and then stimulated with 250 ng/ml biotinylated LPS [40] or 250 ng/ml LPS [17, 18], for 30 min. Protein extracts were prepared as described previously [27]. The samples were immunoprecipitated with mouse anti-GFP antibody for 16–18 h at 4 °C. The recovered proteins were resolved using 10% SDS-PAGE and electrotransferred to a nitrocellulose membrane. Membrane was blocked with 3% bovine serum albumin for 1 h at room temperature and then exposed to streptavidin-HRP (Cell Signaling Technology, Beverly, MA, USA) for 1 h or probed overnight with rabbit anti-GFP antibody, followed by HRP-conjugated secondary antibodies for 1 h, to examine the interference of CPFX and LVFX with LPS binding to TLR4–MD-2 complex. To examine dimerization of TLR4–MD-2 complex, the membrane was probed overnight with either mouse anti-Flag antibody or rabbit anti-GFP antibody. Thereafter, the membrane was exposed to HRP-conjugated secondary antibody for 1 h. Reactive bands were visualized with the ECL substrate (GE Healthcare Amersham, UK), using the VersaDoc Imaging System (Bio-Rad Laboratories, USA) [41]. Densitometry quantification of bands was performed with ImageJ software and biotinylated-LPS or TLR4-Flag expression was normalized to TLR4-GFP levels. Results are expressed as percentage relative to LPS-stimulated cells.

### Statistical analysis

All data represent the results of at least three independent experiments. Statistical analysis was performed using GraphPad Prism Software, version 6.0 (GraphPadSoftware, Inc., San Diego, CA, USA). Results are expressed as mean ± SEM. Data were analyzed by the Kruskal-Wallis nonparametric test followed by Dunn’s post-hoc test for multiple comparisons. A value of *p* < 0.05 was considered to indicate statistically significant differences. Additional details are provided in the figure legends, where appropriate.

## Results

### Binding modes of fluoroquinolones to TLR4–MD-2 complex

**Fig. 1:**
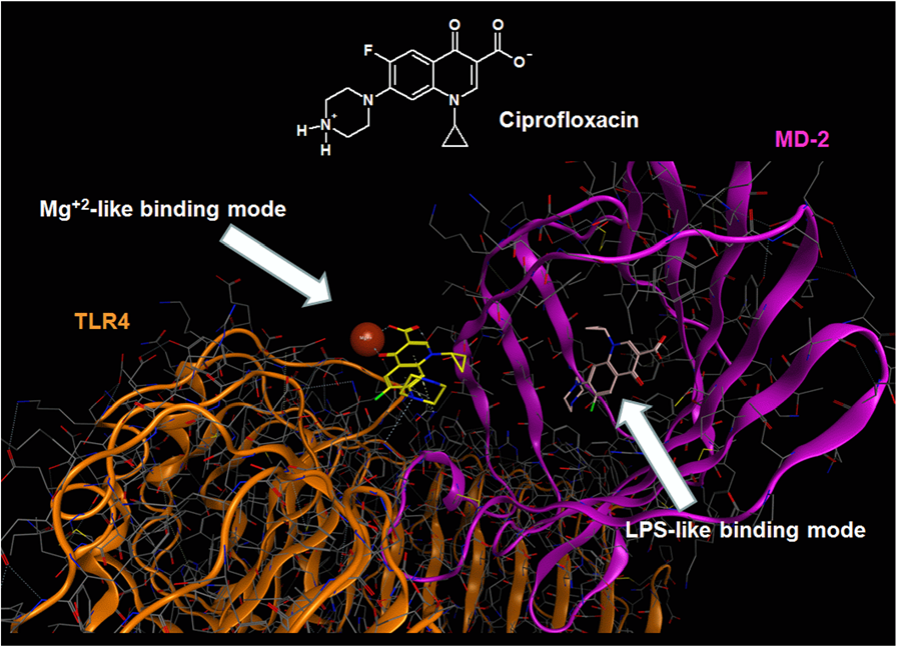
Molecular docking analysis of the two alternative binding modes between ciprofloxacin and TLR4–MD-2 complex. All atoms are voluntarily not showed. TLR4 (colored in orange) and MD-2 (colored in magenta) are represented showing their secondary structure. Mg^2+^ ion is represented by CPK (Corey, Pauling and Koltun model) and is colored in bronze

A molecular docking study was carried out to characterize the putative binding mode to TLR4–MD-2 complex of five largely prescribed FQs, CPFX, LVFX, MXFX, OFX, and DLFX. Interestingly, and similarly to what has already been described for curcumin and its analogues [18], the five docked FQs showed the propensity to bind the TLR4–MD-2 complex in two different modes: occupying the canonical LPS recognition site in the MD-2 structure, and binding at the interface between MD-2 and TLR4 complex where a Mg^2+^ ion is coordinated by LPS, as detected in the crystallographic structure coded by PDB as 3FXI [10, 18]. Considering the analogies in the binding modes of all the docked FQs, the CPFX structure was used as an example for a more detailed description of the two binding modes with the TLR4–MD-2 complex. Firstly, as shown in Fig. 1 and in Additional file 1: Figure S1, CPFX in its zwitterionic form could be accommodated into the large binding pocket of MD-2, occupying a relevant portion of the LPS binding site and assuming different binding modes. Among all generated binding modes of each ligand, the energetically more stable were those that showed important interactions to MD-2, such as charge-charge interactions or hydrogen bonding, engaged with residues Arg90, Glu92, and Tyr102. Secondly, CPFX in its zwitterionic form can also coordinate the Mg^2+^ ion through its carbonyl and carboxyl groups in neighboring positions. The Mg^2+^ coordination could prevent the stabilization of the LPS binding to MD-2, interfering with the TLR4 dimerization process. Based on the in silico analysis, the two most popular FQs CPFX and LVFX were used with the aim of characterizing the molecular mechanism involved in the regulation of microglia inflammatory response.

### Determination of non-cytotoxic concentrations of ciprofloxacin and levofloxacin in microglial cells

**Fig. 2:**
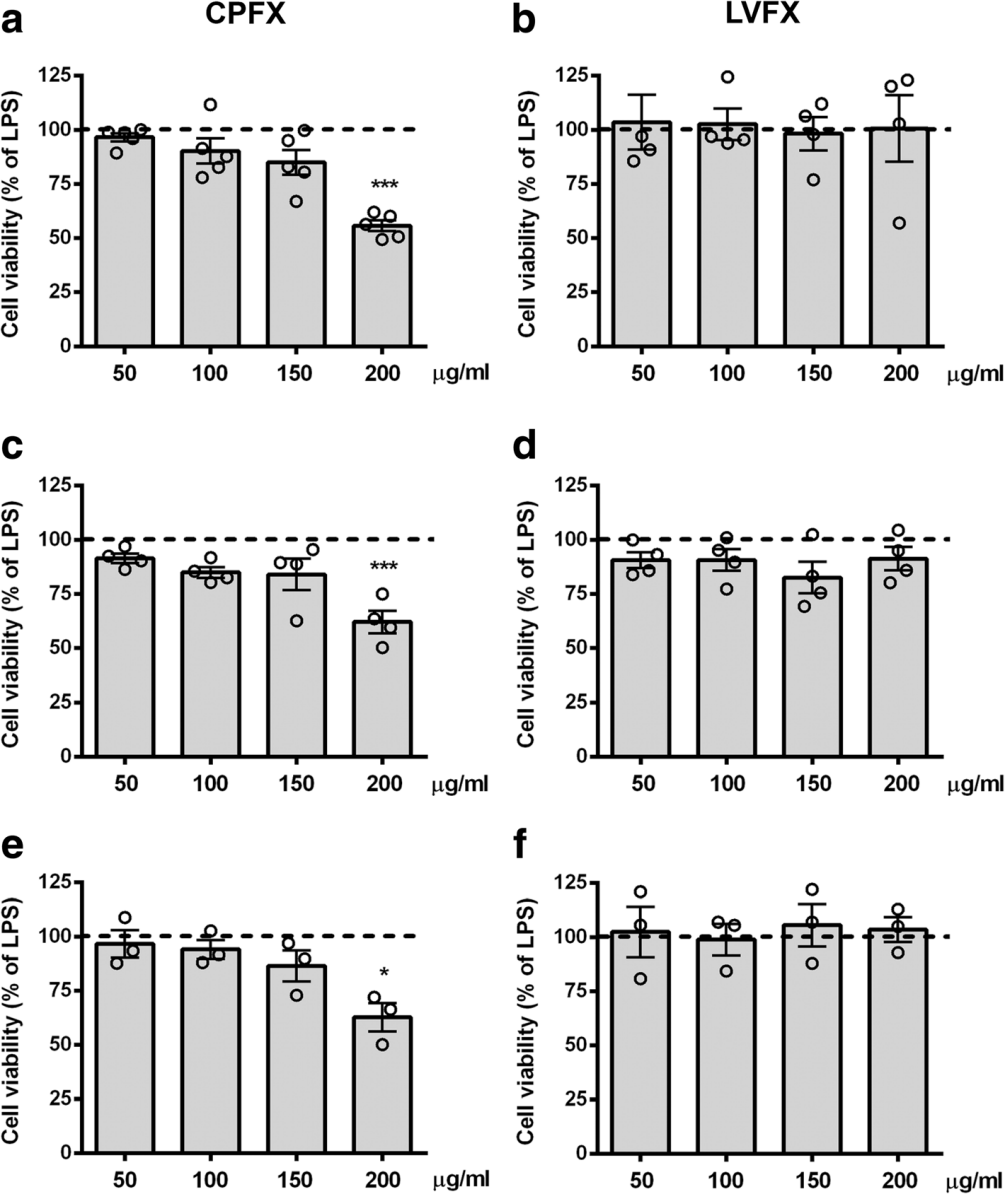
Effects of ciprofloxacin and levofloxacin in microglia cell viability. Microglia were cultured for 24 h in 10% serum-containing medium, which was replaced with serum-free medium before pre-treatment with (**a**, **c**, **e**) ciprofloxacin (CPFX) and (**b**, **d**, **f**) levofloxacin (LVFX) (50–200 μg/ml) for 1 h followed by stimulation with 100 ng/ml LPS for 6 h. At the end of incubation, cell viability was determined by (**a**, **b**) MTT, (**c**, **d**) SRB, and (**e**, **f**) Trypan blue exclusion assays. Results are expressed as percentage of cell viability relative to LPS-stimulated microglia. Data are means ± SEM (*n* = at least three independent experiments). Data were analyzed by Kruskal-Wallis test (*p =* 0.0020 and *p =* 0.8929 in **a**, **b**; *p =* 0.0048 and *p =* 0.3920 in **c**, **d**; *p =* 0.0376 and *p =* 0.9245 in **e**, **f**), followed by Dunn’s multiple comparison test. *p < 0.05 and ***p < 0.001 versus LPS-stimulated cells (dashed lines)

We first performed experiments to examine the safety, and identify a non-cytotoxic concentration range, of CPFX and LVFX in microglia using three different viability assays (i.e., MTT, SRB, and Trypan blue exclusion tests). Cultures were serum starved for 2 h and then incubated with increasing concentrations (50–200 μg/ml) of the two drugs applied alone or in the presence of LPS stimulation for 6 h. Cell viability of cultures exposed to CPFX at concentrations higher than 150 μg/ml significantly decreased compared to LPS-activated cultures, taken as 100% (Fig. 2a, c, and e). In contrast, LVFX did not show any cytotoxic effects at the concentrations tested (Fig. 2b, d, and f). Similar results were obtained in LPS-unstimulated control microglia (data not shown). Based on these results, concentrations of the two drugs used in the following studies ranged from 50 to 150 μg/ml.

### Ciprofloxacin and levofloxacin inhibit LPS-induced pro-inflammatory response in microglial cells

**Fig. 3:**
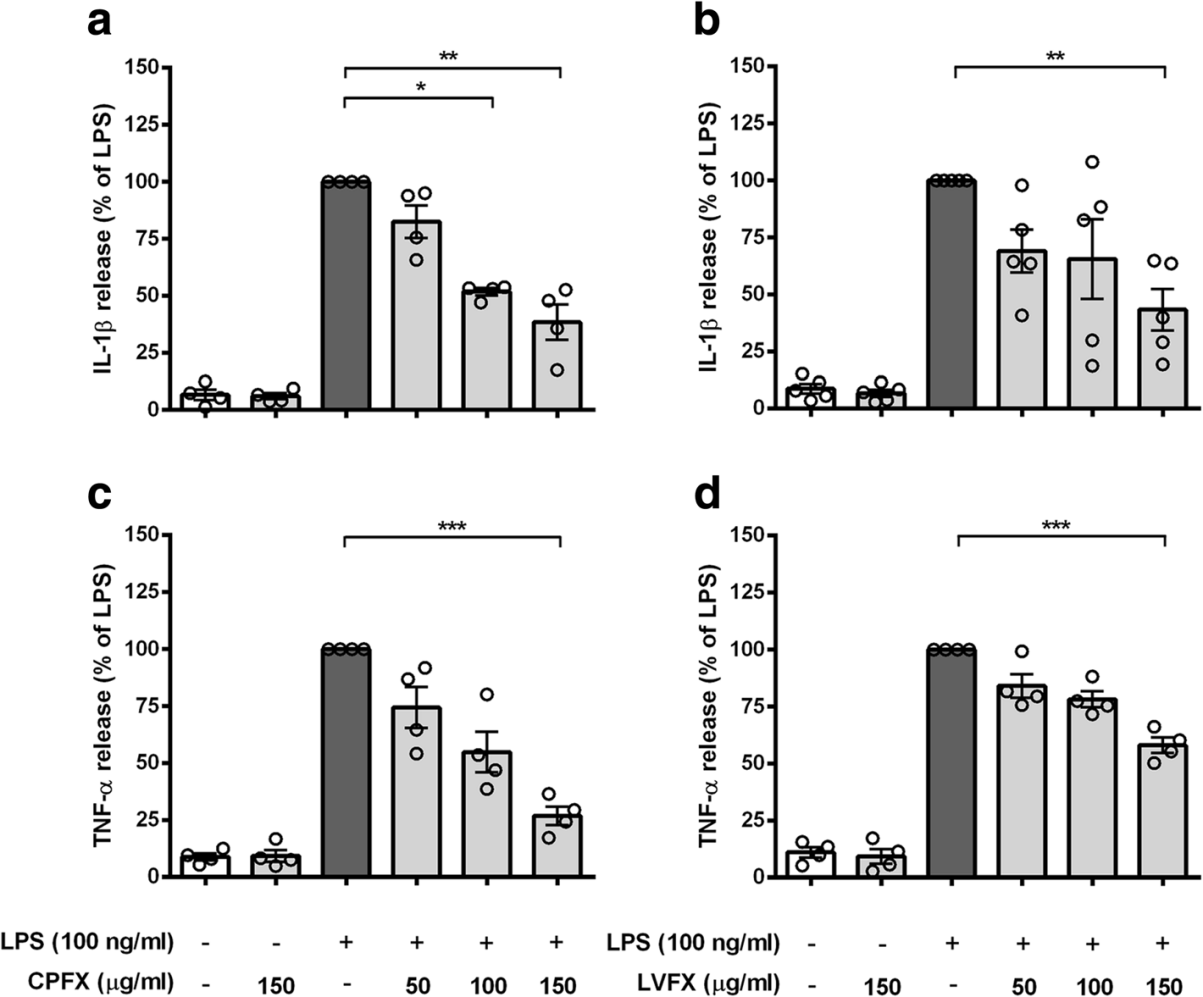
Effects of ciprofloxacin and levofloxacin on cytokine release from LPS-stimulated cortical microglia. Microglia were subcultured for 24 h in 10% FBS-containing medium, which was replaced with serum-free medium before pretreatment with (**a**, **c**) ciprofloxacin (CPFX) and (**b**, **d**) levofloxacin (LVFX) (50–150 μg/ml) for 1 h followed by stimulation with 100 ng/ml LPS for 6 h. Supernatants were collected and analyzed for (**a**, **b**) IL-1β and (**c**, **d**) TNF-α release. Results are expressed as percentage of cytokine release relative to LPS-stimulated microglia (dark gray bars). Data are means ± SEM (*n* = at least four independent experiments). Data were analyzed by Kruskal-Wallis test (*p* < 0.0001 and *p =* 0.0191 in **a**, **b**; *p* < 0.0001 in **c**, **d**) followed by Dunn’s multiple comparison test. *p < 0.05, **p < 0.01, and ***p < 0.001 versus LPS stimulation

In addition to their antimicrobial activity, many FQs exert a significant immunomodulatory effect in peripheral immune cells [21–25]. We examined the possible anti-inflammatory effect of CPFX and LVFX in an in vitro model of neuroinflammation. Microglial cells were exposed for 1 h to concentrations of CPFX or LVFX not affecting cell viability (50–150 μg/ml; Fig. 2) and then stimulated with LPS for 6 h to induce an inflammatory response. Concentrations of IL-1β and TNF-α released in the culture supernatants were measured by ELISA. As expected, the release of IL-1β and TNF-α was dramatically increased in response to LPS stimulation compared to the control cells. This effect was significantly suppressed by pretreatment with the two FQs. The two drugs themselves had no effect on the low amounts of IL-1β and TNF-α released by unstimulated microglia (Fig. 3). These results provide previously unavailable evidence, to the best of our knowledge, that CPFX and LVFX exhibit anti-inflammatory activity on an in vitro model of CNS inflammation.

### Ciprofloxacin and levofloxacin inhibit LPS-induced NF-κB activation in microglial cells

**Fig. 4:**
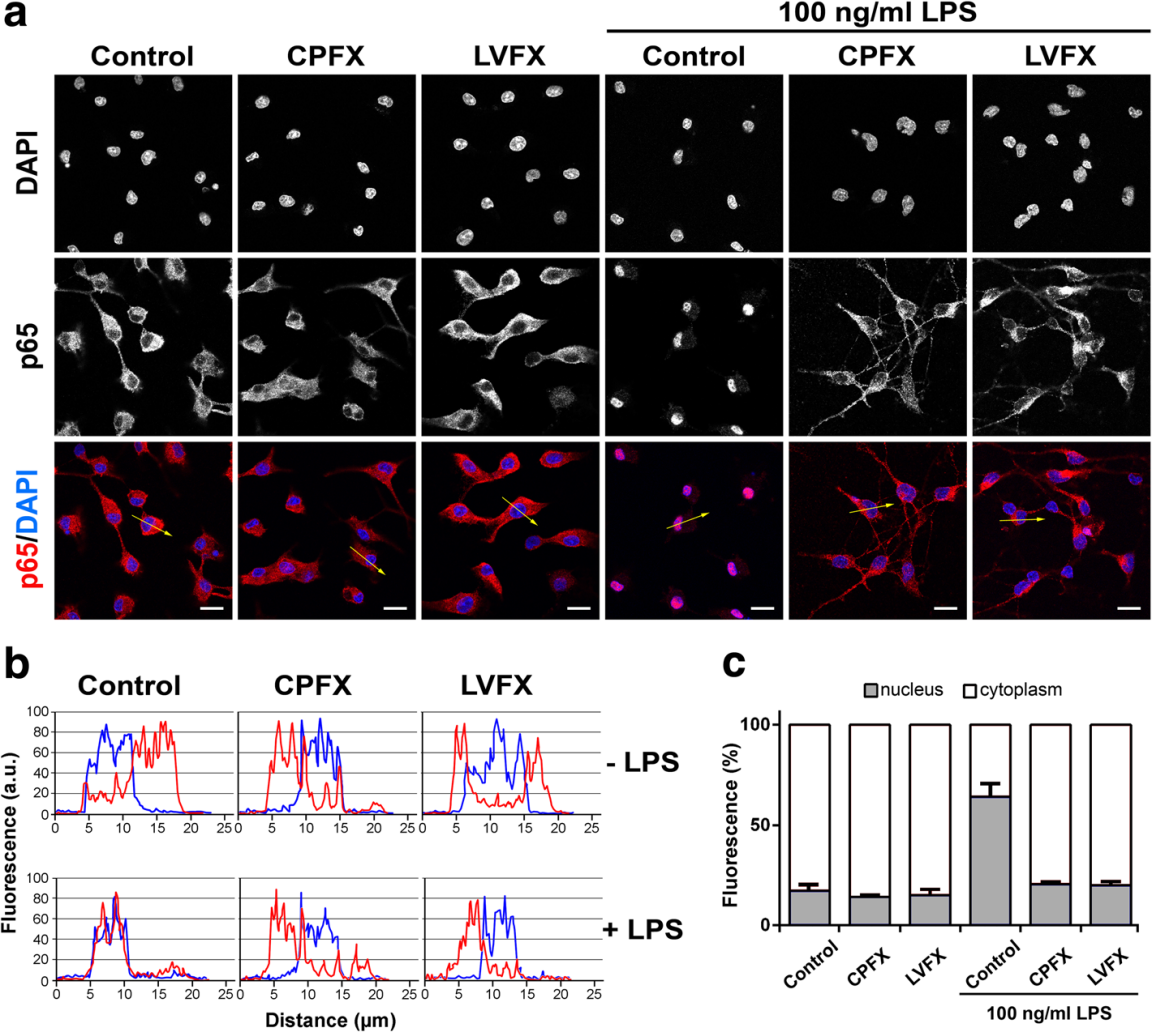
Effects of ciprofloxacin and levofloxacin on NF-κB activation in unstimulated and LPS-stimulated microglia. Cells were subcultured for 24 h in 10% serum-containing medium, which was replaced with serum-free medium before stimulation with ciprofloxacin (CPFX) and levofloxacin (LVFX) (500 μg/ml) ± 100 ng/ml LPS. **a** Cells were processed for NF-κB p65 immunostaining. Experiments were performed three times and representative confocal images showing subcellular localization of p65 are shown. Scale bars, 10 μm. **b** Fluorescence intensity profiles along an ideal oriented line across a representative cell (yellow arrows in the third line of (**a**)). **c** The fluorescence intensity of cytoplasmic and nuclear p65 subunit was calculated using ImageJ software and results are presented as a percentage of nuclear (gray bars) over cytoplasmic NF-κB p65 (white bars). Data are mean ± SEM from five random fields of three separate experiments

In the attempt to define the underlying molecular mechanisms by which the two FQs suppressed microglia inflammatory response, we monitored the activation of the transcription factor NF-κB, which occurs in response of pro-inflammatory stimuli and results in increased expression of many cytokines and chemokines [42]. To this end, we quantified nuclear translocation of NF-κB p65 subunit as an indicator of NF-κB activation. Neither CPFX nor LVFX alone induced NF-κB activation, as shown by a predominantly cytoplasmic distribution of p65 subunit, similar to that observed in untreated control cells (Fig. 4a, column 1, 2, and 3; Fig. 4b, upper graphs; and Fig. 4c, bar 1, 2, and 3). As previously shown, LPS exposure induced a significant translocation of p65 subunit from the cytosol to the nucleus (Fig. 4a–c, column, graph, and bar 4), indicative of NF-κB activation [18]. In contrast, pretreatment with CPFX and LVFX suppressed the LPS-induced p65 nuclear translocation (Fig. 4a–c, column, graph, and bar 5 and 6), suggesting that inhibition of NF-κB activation may contribute to the anti-inflammatory effect of the two quinolone derivatives.

### Ciprofloxacin and levofloxacin prevent MD-2-LPS interaction and TLR4–MD-2 dimerization

**Fig. 5:**
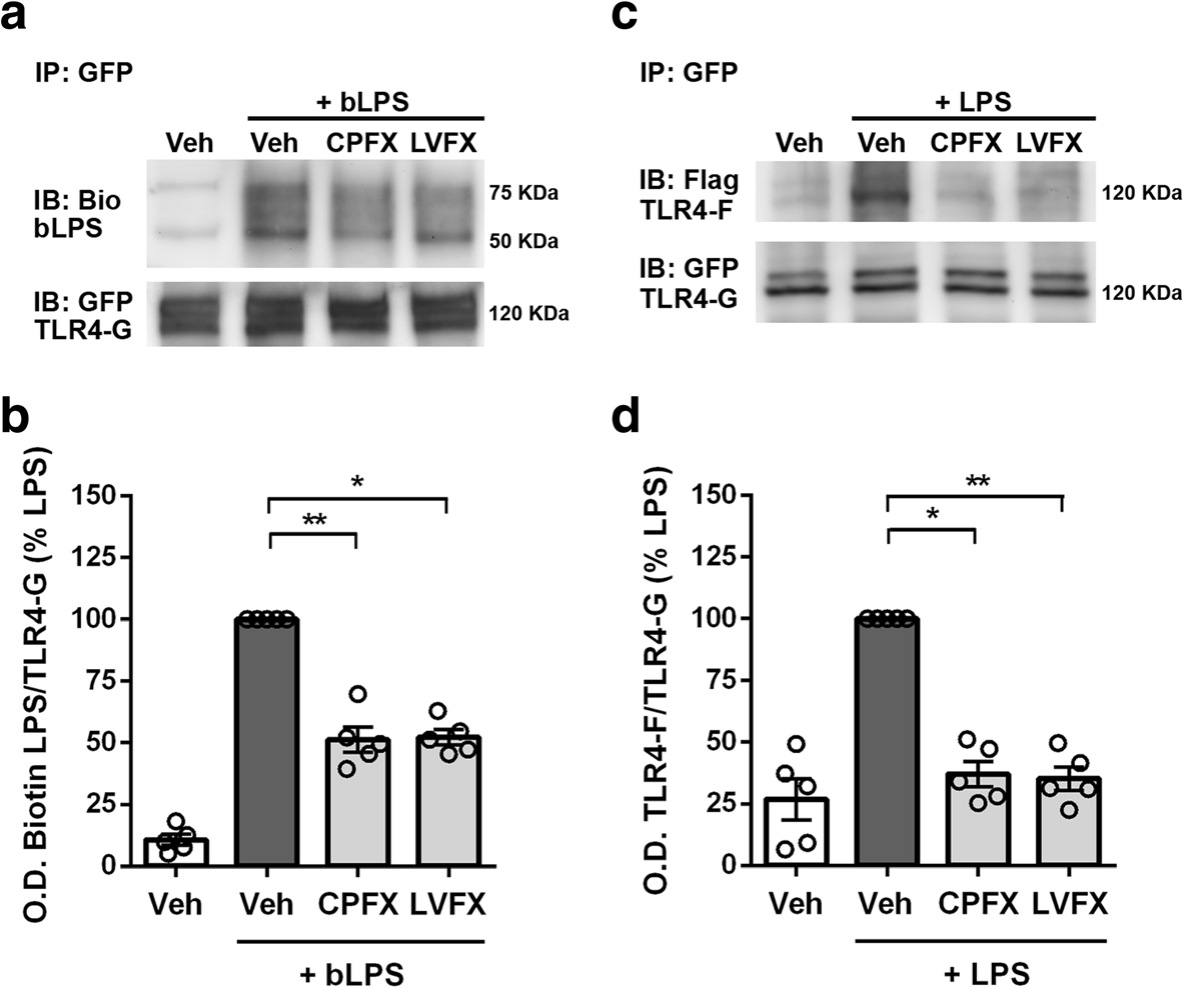
Effects of ciprofloxacin and levofloxacin on LPS binding and LPS-induced TLR4 dimerization. Ba/F3 cells expressing TLR4-Flag (TLR4-F), TLR4-GFP (TLR4-G), MD2-Flag, and CD14 were pretreated with 500 μg/ml ciprofloxacin (CPFX) or levofloxacin (LVFX) for 1 h and then stimulated with 250 ng/ml of **a** biotinylated LPS (bLPS) or **c** LPS for 30 min. Cell lysates were immunoprecipitated with mouse anti-GFP antibody. The immunoblotted proteins were exposed to **a** streptavidin-HRP (upper) or chicken anti-GFP antibody (lower), and the ratio of Biotin-LPS/TLR4-GFP is shown in (**b**); **c** mouse anti-Flag (upper) or chicken anti-GFP (lower) antibodies and the ratio of TLR4-Flag/TLR4-GFP is shown in (**d**). Experiments were performed five times and representative images are shown. Data, expressed as percentage of LPS-stimulated cells, are shown as means ± SEM (*n* = 5). Data were analyzed by Kruskal-Wallis test (*p* = 0.0021) followed by Dunn’s multiple comparison test. *p < 0.05 and **p < 0.01 versus LPS stimulation. *bLPS* biotinylated LPS, *Bio* biotin, *IP* immunoprecipitation, *IB* immunoblot, *Veh* vehicle

Lastly, to identify the target of CPFX and LVFX at receptor level, we investigated the involvement of TLR4 in the signaling pathway underlying the anti-inflammatory effect of the two antibiotics. LPS binding to MD-2 and the following TLR4–MD-2 complex dimerization are the initial steps involved in the activation of inflammatory response mediated by TLR4 signaling pathway [10, 43]. To address the question of whether CPFX and LVFX affected the proper assembly of TLR4–MD-2–LPS ternary complex on the cell surface, we first co-immunoprecipitated biotinylated LPS in Ba/F3 cells expressing human TLR4-GFP, TLR4-Flag, MD-2-Flag, and CD14 [27]. Cells were preincubated with CPFX and LVFX for 1 h prior to stimulation with biotinylated LPS for 30 min. To detect LPS binding, TLR4-GFP was immunoprecipitated and co-precipitation of biotinylated LPS was probed with streptavidin-HRP conjugated and determined by immunoblot analysis. While CPFX and LVFX did not affect precipitation of TLR4-GFP (Fig. 5a, bottom lanes), they attenuated the association of biotinylated LPS with TLR4–MD-2 complex (Fig. 5a, b).

Next, we examined whether the two drugs affected LPS-induced TLR4–MD-2 dimerization using the same cell line. After pretreatment with CPFX and LVFX and LPS stimulation, TLR4-GFP was immunoprecipitated with anti-GFP antibody and co-precipitation of TLR4-Flag was probed with anti-Flag antibody. The dimerization observed after LPS treatment was reduced by CPFX and LVFX treatment (Fig. 5c, d). All of the treatments did not affect precipitation of TLR4-GFP (Fig. 5c, bottom lanes). Taken together, these results indicate that the two FQs tested in this study exhibit their effect on LPS-induced microglia inflammatory response through targeting LPS binding to TLR4–MD-2 complex and the subsequent receptor dimerization, resulting in downregulation of the ensuing intracellular signaling.

## Discussion

We recently hypothesized that curcumin and some of its analogues can interact with the TLR4–MD-2 complex in two different modes: first, by occupying the canonical LPS’s recognition site in the MD-2 structure, and second, by binding at the interface between MD-2 and TLR4 complex where a magnesium ion is coordinated by LPS [10, 18]. We have also argued that the second Mg^2+^-driven binding site is accessible only if the ligand is able to appreciably coordinate the metal ion, as it occurs, for example, for curcumin and its analogues that maintain the 1,3-diketone moiety, as well as their enolate form. Based on these observations, we explored if other chemical structures containing putative Mg^2+^ chelating groups would also display an efficient ability to inhibit the signal pathway activated by LPS and mediated by TLR4. As anticipated, one of the most important class of drugs containing a putative Mg^2+^ chelating group is certainly that of quinolones [44]. The central bicyclic ring of quinolones and their fluorinated derivatives has a keto-carbonyl group at positions 3 and 4 that efficiently binds divalent ions, such as Mg^2+^, Ca^2+^, Cu^2+^, Zn^2+^, Fe^2+^, Co^2+^, forming chelates with 1:1 or 1:2 (metal:ligand) stoichiometry where the carbonyl and carboxyl groups in neighboring positions are the most common coordination mode in the quinolone chelates [45, 46]. These drugs are one of the most important classes of antibiotics identified in the past 50 years, and more than 1000 analogues have since been synthesized and evaluated in an attempt to reduce toxicity and increase antimicrobial potency. In particular, FQs are an important subclass of potent quinolones with a broad spectrum of activity against gram-(+), gram-(−) and mycobacterial organisms as well as anaerobes, thus offering great therapeutic potential, particularly against those organisms that resist to other classes of antibacterial drugs [47]. Furthermore, in addition to their antimicrobial properties, FQs exert direct immunomodulatory and anti-inflammatory activities, resulting in beneficial effects in various inflammatory conditions. These findings led us to hypothesize that, due to their chemical structure and properties, FQs could exert anti-inflammatory activities by affecting the proper assembly of the TLR4–MD-2–LPS ternary complex, whose activation is associated with a number of pathologies, including CNS diseases. Molecular docking studies showed that five of the commonly prescribed FQs (i.e., CPFX, LVFX, MXFX, OFX, and DLFX) are potentially able to interfere with the correct assembly of the TLR4–MD-2–LPS ternary complex, through the same dual mechanism recently proposed also for curcumin and its analogues [18].

Starting from this in silico hypothesis, we have shown for the first time that the two FQs CPFX and LVFX decrease the in vitro release of IL-1β and TNF-α by LPS-stimulated primary microglia. This effect was achieved at concentrations of CPFX (100 μg/ml) and LVFX (150 μg/ml) higher than clinically relevant concentrations, but in agreement with some previous studies conducted in peripheral immune cells aimed at exploring the anti-inflammatory properties of these drugs [23, 48, 49]. However, it should be emphasized that in our experimental conditions, the FQ concentrations used failed to produce any cytotoxic effect on microglia. This was assessed using three different viability assays (i.e., MTT, SRB, and Trypan blue assays) in order to avoid the possible interference of metabolic activity of LPS-activated microglia with the MTT assay, which might cause an overestimation of cell number [50]. Results from SRB assay, which is based on the ability of SRB to bind to protein components of cells [51], and from Trypan blue exclusion assay, based on the principle that cell membranes of viable cells are impermeable to the dye [39], correlated well with those obtained with the MTT assay, confirming that the viability observed was not affected by metabolic artifacts.

Although the mechanisms of antibacterial activity of FQs have been extensively investigated in vitro and in vivo, data on the precise molecular mechanisms underlying their immunomodulatory activity are still lacking. Previous studies have shown that FQ treatment results in the downregulation of pro-inflammatory cytokines, inducible nitric oxide synthase and cyclooxygenase-2, and the upregulation of IL-10 expression [21, 22, 52]. However, at present, it is still unclear which transcription factors mediate these effects. One of the key factors that regulate a variety of genes involved in different processes of the inflammatory response is the transcription factor NF-κB, which controls the final immune response via mRNA expression of an array of inflammatory cytokines and chemokines [42, 53]. In resting cells, NF-κB is bound to an inhibitory protein, inhibitory-κB, and retained in the cytoplasm. Cell stimulation with different cues (e.g., LPS) triggers signal transduction pathways that ultimately result in nuclear translocation of NF-κB p65 component, which contains the main transcriptional regulatory domain responsible for the activation of NF-κB responsive genes [54]. In microglia cells, decreased release of IL-1β and TNF-α mediated by CPFX and LVFX was associated with significant inhibition of NF-κB nuclear translocation, confirming that the two drugs interfere with a crucial regulatory factor implicated in the production of inflammatory cytokines. NF-κB pathway is triggered by several stimuli, most important of which is the activation of TLRs, including TLR4 [54]. Based on our in silico observation suggesting interference with the correct assembly of the TLR4–MD-2–LPS ternary complex, we examined whether CPFX and LVFX could act at receptor level, using Ba/F3 cells expressing human TLR4, MD-2, and CD14. Ba/F3 cells have a rapid cellular proliferation rate, which makes them a valuable tool for immunoprecipitation studies. Furthermore, Ba/F3 cells used in this study stably express TLR4–MD-2 complex differently tagged (i.e., GFP and Flag), which allowed us to easily study LPS interaction with TLR4 and receptor dimerization, avoiding transfection of primary microglia. In fact, traditional methods of microglia transfection, such as chemical-based methods, viral transduction, and electroporation, exhibit low transfection efficiency, affect cell survival, and promote inflammatory responses [55]. Furthermore, the induction of TLR4 dimerization by LPS was already shown in this cell line [18, 27]. Co-immunoprecipitation studies demonstrated that CPFX and LVFX interfere with the LPS-induced inflammatory response by inhibiting TLR4–MD-2 dimerization as well as LPS binding to the TLR4–MD-2 complex, validating molecular docking observations: CPFX and LVFX interact with LPS or MD-2 to prevent LPS binding to TLR4–MD-2.

## Conclusions

**Fig. 6:**
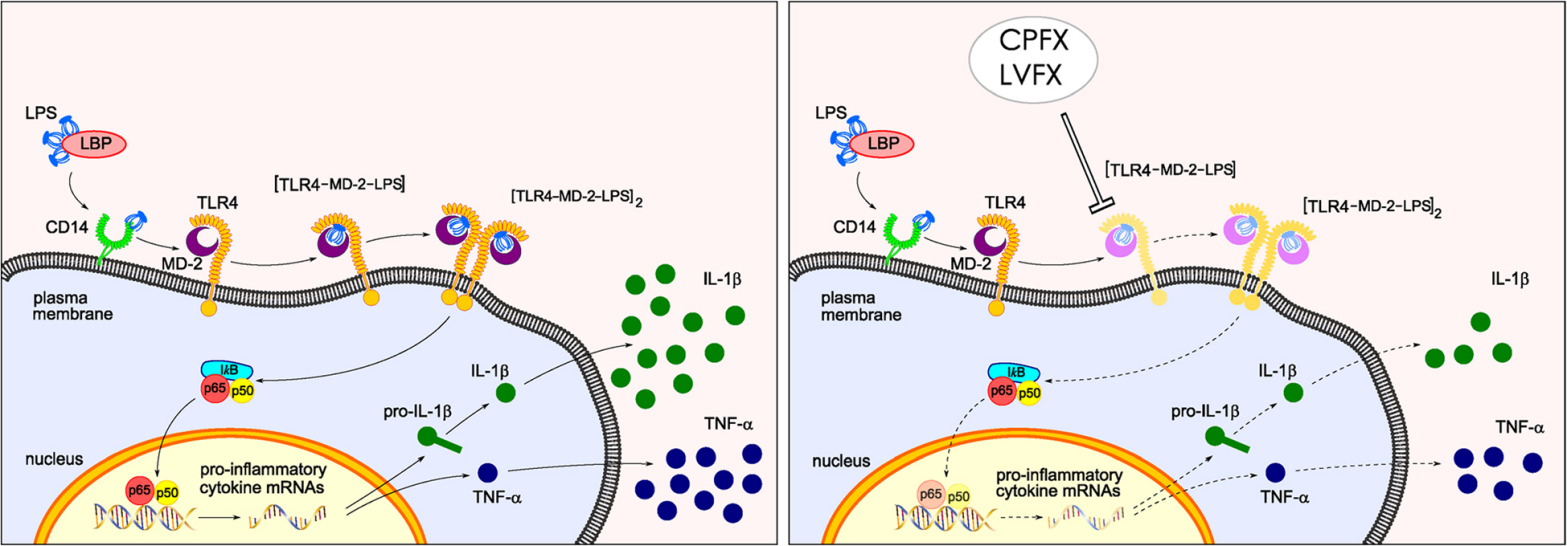
Model depicting cascade of the anti-inflammatory effect of ciprofloxacin and levofloxacin, targeting TLR4–MD-2 complex. LBP facilitates transfer of LPS monomers to CD14, which subsequently shifts the endotoxin to TLR4/MD-2 complex, then leading to formation of TLR4/MD2/LPS ternary complex and its subsequently dimerization. Dimerization of the receptor complex induces the activation of intracellular signaling pathways that involve NF-κB activation and lead to production of pro-inflammatory cytokines (left panel). Fluoroquinolones, such as CPFX and LVFX, exert anti-inflammatory activity, through the inhibition of TLR4/MD2/LPS ternary complex formation, receptor dimerization, and NF-κB nuclear translocation (depicted in light colors in the right panel), resulting in a decreased production of pro-inflammatory cytokines. *CD14* cluster of differentiation 14, *CPFX* ciprofloxacin, *IL*-*1β* interleukin-1 β, *LBP* LPS binding protein, *LPS* lipopolysaccharide, *LVFX* levofloxacin, *MD*-*2* myeloid differentiation protein-2, *TLR4* Toll-like receptor 4, *TNF*-*α* tumor necrosis factor-α

In this study, we characterized a mechanism that could explain the anti-neuroinflammatory effect of CPFX and LVFX. We showed that TLR4–MD-2 complex is the primary target of the anti-inflammatory activity of the two drugs and that the two antibiotics decreased the binding of LPS to MD-2, resulting in the downregulation of LPS-induced inflammatory response, via TLR4–MD-2/NF-κB pathway (Fig. 6). Although this signaling pathway has been widely associated with the neuroinflammatory response [56, 57], this study shows for the first time that CPFX and LVFX exert anti-inflammatory activity, at least in part, through modulation of TLR4-MD2/NF-κB signaling. These results provide further evidence that molecules, such as FQs, capable of interfering with LPS binding to MD-2 could be useful leads to design and develop novel agents that prevent LPS-mediated TLR4 activation.

## Additional files

Additional file 1: **Figure S1.** Starting from the overall structure of the human TLR4–MD-2 complex derived by the crystallographic structure 3FXI, the two putative binding sites of docked ciprofloxacin are zoomed and shown as Connolly surface. Hydrogen atoms are voluntarily omitted. Hydrophobic regions of the Connolly surface are colored in green, polar in magenta and the exposed regions of the surface to the solvent in red. Mg^2+^ ion is represented by CPK and is colored in bronze. (JPG 149 kb)

## Data Availability

The datasets generated and/or analyzed during the current study are available from the corresponding author on reasonable request.

## Abbreviations

ANOVA: One-way analysis of variance
CNS: Central nervous system
CPFX: Ciprofloxacin
CPK: Corey, Pauling, and Koltun model
DAPI: 4,6-Diamidino-2-phenylindole
DLFX: Delafloxacin
DMEM: Dulbecco’s modified eagle medium
FCS: Fetal calf serum
FQs: Fluoroquinolones
HRP: Horseradish peroxidase
IL: Interleukin
LPS: Lipopolysaccharide
LVFX: Levofloxacin
MD-2: Myeloid differentiation protein-2
MOE: Molecular operation environment program
MTT: 3-(4,5-Dimethylthiazol-2-yl)-2,5-diphenyltetrazolium bromide
MXFX: Moxifloxacin
NF: Nuclear factor
OFX: Ofloxacin
PDB: Protein data bank
rms: Root-mean-square
SRB: Sulforhodamine B
TLR: Toll-like receptor
TNF-α: Tumor necrosis factor-alpha
