# Supplementary figures and images for: Ciprofloxacin and levofloxacin attenuate microglia inflammatory response via TLR4/NF-kB pathway
Source: J Neuroinflammation. 2019 Jul 18;16:148. doi: 10.1186/s12974-019-1538-9 (PMC6637517; doi:10.1186/s12974-019-1538-9)

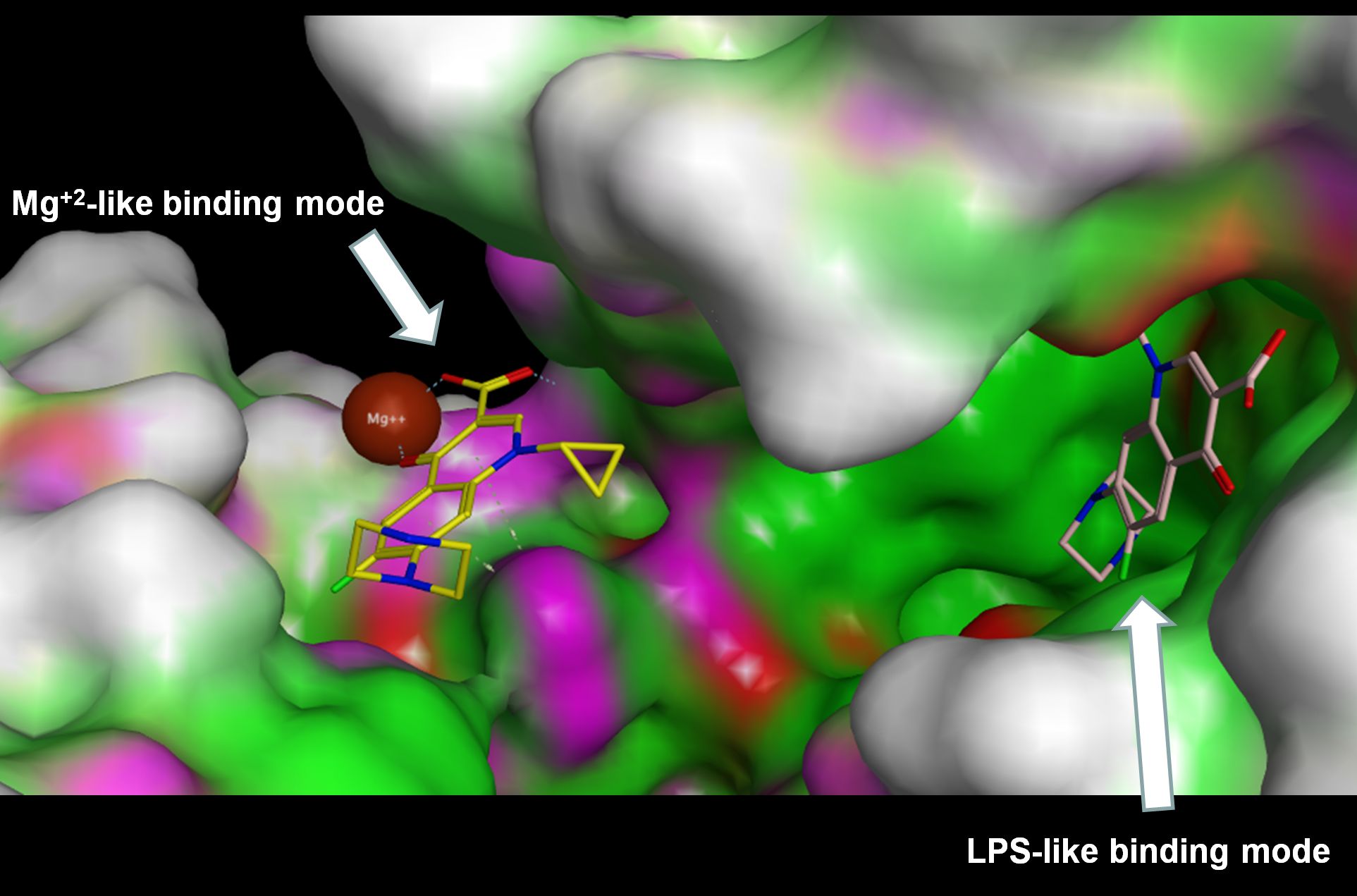

Supplement: Supplementary file 1 — Figure S1. Starting from the overall structure of the human TLR4–MD-2 complex derived by the crystallographic structure 3FXI, the two putative binding sites of docked ciprofloxacin are zoomed and shown as Connolly surface. Hydrogen atoms are voluntarily omitted. Hydrophobic regions of the Connolly surface are colored in green, polar in magenta and the exposed regions of the surface to the solvent in red. Mg2+ ion is represented by CPK and is colored in bronze. (JPG 149 kb) [file 12974_2019_1538_MOESM1_ESM.jpg]
